# Investigating the Cost-Effectiveness of Telemonitoring Patients With Cardiac Implantable Electronic Devices: Systematic Review

**DOI:** 10.2196/47616

**Published:** 2024-04-19

**Authors:** Sarah Raes, Andrea Prezzi, Rik Willems, Hein Heidbuchel, Lieven Annemans

**Affiliations:** 1 Department of Public Health and Primary Care Ghent University Gent Belgium; 2 Department of Cardiovascular Sciences Universiteit Leuven Leuven Belgium; 3 Department of Genetics, Pharmacology and Physiopathology of Heart, Blood Vessels and Skeleton (GENCOR) Antwerp University Antwerp Belgium

**Keywords:** systematic review, cost-effectiveness, telemonitoring, cardiac device, implantable cardioverter-defibrillator, ICD, pacemaker, monitoring, patient management, effectiveness, cost, quality of life, cardiac implantable electronic device, cardiac

## Abstract

**Background:**

Telemonitoring patients with cardiac implantable electronic devices (CIEDs) can improve their care management. However, the results of cost-effectiveness studies are heterogeneous. Therefore, it is still a matter of debate whether telemonitoring is worth the investment.

**Objective:**

This systematic review aims to investigate the cost-effectiveness of telemonitoring patients with CIEDs, focusing on its key drivers, and the impact of the varying perspectives.

**Methods:**

A systematic review was performed in PubMed, Web of Science, Embase, and EconLit. The search was completed on July 7, 2022. Studies were included if they fulfilled the following criteria: patients had a CIED, comparison with standard care, and inclusion of health economic evaluations (eg, cost-effectiveness analyses and cost-utility analyses). Only complete and peer-reviewed studies were included, and no year limits were applied. The exclusion criteria included studies with partial economic evaluations, systematic reviews or reports, and studies without standard care as a control group. Besides general study characteristics, the following outcome measures were extracted: impact on total cost or income, cost or income drivers, cost or income drivers per patient, cost or income drivers as a percentage of the total cost impact, incremental cost-effectiveness ratios, or cost-utility ratios. Quality was assessed using the Consensus Health Economic Criteria checklist.

**Results:**

Overall, 15 cost-effectiveness analyses were included. All studies were performed in Western countries, mainly Europe, and had primarily a male participant population. Of the 15 studies, 3 (20%) calculated the incremental cost-effectiveness ratio, 1 (7%) the cost-utility ratio, and 11 (73%) the health and cost impact of telemonitoring. In total, 73% (11/15) of the studies indicated that telemonitoring of patients with implantable cardioverter-defibrillators (ICDs) and cardiac resynchronization therapy ICDs was cost-effective and cost-saving, both from a health care and patient perspective. Cost-effectiveness results for telemonitoring of patients with pacemakers were inconclusive. The key drivers for cost reduction from a health care perspective were hospitalizations and scheduled in-office visits. Hospitalization costs were reduced by up to US $912 per patient per year. Scheduled in-office visits included up to 61% of the total cost reduction. Key drivers for cost reduction from a patient perspective were loss of income, cost for scheduled in-office visits and transport. Finally, of the 15 studies, 8 (52%) reported improved quality of life, with statistically significance in only 1 (13%) study (*P*=.03).

**Conclusions:**

From a health care and patient perspective, telemonitoring of patients with an ICD or a cardiac resynchronization therapy ICD is a cost-effective and cost-saving alternative to standard care. Inconclusive results were found for patients with pacemakers. However, telemonitoring can lead to a decrease in providers’ income, mainly due to a lack of reimbursement. Introducing appropriate reimbursement could make telemonitoring sustainable for providers while still being cost-effective from a health care payer perspective.

**Trial Registration:**

PROSPERO CRD42022322334; https://tinyurl.com/puunapdr

## Introduction

### Background

The implantation rates of cardiac implantable electronic devices (CIEDs), including pacemakers and implantable cardioverter-defibrillators (ICDs), have increased over the last decades due to expanded indications and a progressively aging population [1]. To evaluate the clinical status of the patient and device functioning, current guidelines recommend that older patients with pacemakers should be evaluated every 3 to 12 months and patients with ICDs should be evaluated every 3 to 6 months [2]. This regimen imposes a considerable burden on patients and physicians if the patient is required to be seen in person.

Telemonitoring, referring to the process of using telecommunication and information technology to monitor the health status of a patient and device function from a distance, can reduce this burden by replacing some in-office visits with transmissions from the patients’ home [3]. Existing research indicated that telemonitoring is safe (eg, experiencing equal major adverse events to standard care) [4,5]. The advantages of telemonitoring include fewer inappropriate shocks for patients with ICDs [4,6] and fewer hospitalizations for patients with atrial arrhythmias and strokes [4,6,7]. Moreover, there is a rapid detection of cardiovascular events and device malfunction [5,7], leading to a time reduction between clinical decision and intervention [8].

Besides the effectiveness of telemonitoring, patient experience is essential in high-quality health care services. Overall, patients with pacemakers on telemonitoring reported positive experiences comparable to the experience of patients with in-hospital monitoring [9]. Telemonitored patients with pacemakers tended to receive less information about their diagnosis but no significant differences were found in other items, such as confidence in clinicians, treatment decision involvement, treatment satisfaction, and waiting time before admission [9]. Another study indicated that telemonitoring of patients with a cardiac resynchronization therapy defibrillator (CRT-D) was time-saving for both patients and physicians [10].

Cost-effectiveness analyses are important to quantify the value of new interventions, informing both medical decision-making and public policy [11]. However, cost-effectiveness analyses depend on the perspective considered. The different perspectives are the health care payer perspective (eg, Medicare or Medicaid and British National Health Service), the patient perspective, the provider perspective (eg, physician), and the society perspective. The health care payer and societal perspectives differ from each other as the societal perspective includes indirect nonmedical costs (eg, transport) [12].

### Objectives

As cost-effectiveness analyses have shown heterogeneous results, it is still debatable whether telemonitoring is worth the investment relative to standard care. However, data on cost-effectiveness are important for health care payers to make decisions on the reimbursement of telemonitoring. Lack of reimbursement can be an important adoption barrier for new technology [13,14]. For these 2 reasons, this paper reviews the cost-effectiveness of telemonitoring, reviews how the results differ from different perspectives, and describes the key drivers of the cost-effectiveness of telemonitoring.

## Methods

### Overview

The review protocol was published by PROSPERO (International Prospective Register of Systematic Reviews; CRD42022322334). This systematic review was carried out in accordance with the PRISMA (Preferred Reporting Items for Systematic Reviews and Meta-Analyses) reporting guideline of 2020 [15], and the PRISMA-ScR (Preferred Reporting Items for Systematic Reviews and Meta-Analyses extension for Scoping Reviews) [16], which can be found in the Multimedia Appendix 1. Guidelines for preparing a systematic review of health economic evaluations were followed [17].

### Literature Search

For this review, PubMed, Embase, EconLit, and Web of Science Core Collection were systematically searched. The last search was performed on July 7, 2022. No filters (eg, publication date or type of study) were applied. Search strategies for all electronic databases can be found in Multimedia Appendix 2.

Search strings were developed based on explorations of databases and previous reviews. The following key concepts were translated into strings: (1) CIEDs, (2) telemonitoring, and (3) economic evaluations (eg, cost-effectiveness analyses and cost-utility analyses). The latter was based on a validated search filter, designed to identify economic evaluations, and was broadened for this study to maximize sensitivity [18]. The search terms for CIEDs and telemonitoring were based on existing reviews [19-21].

### Study Selection

**Figure 1:**
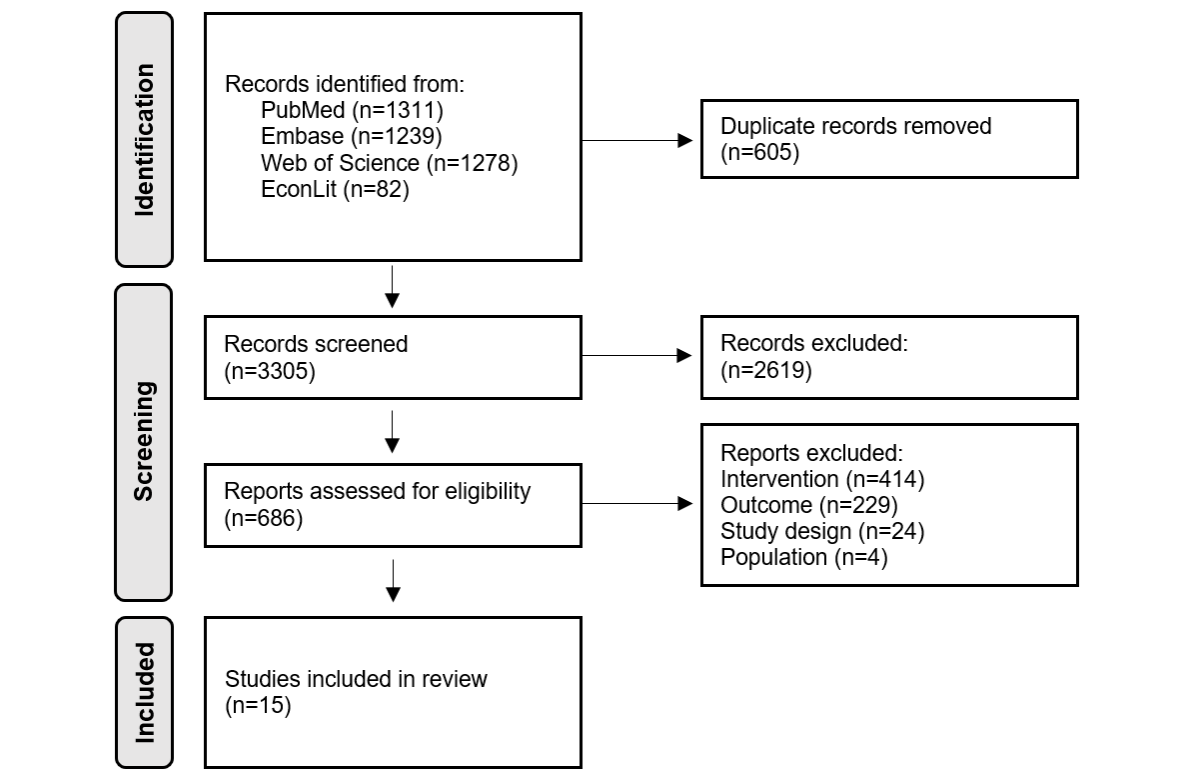
Flowchart of the study selection.

Studies were included if their primary focus was on the cost-effectiveness of telemonitoring patients with a CIED. The eligibility criteria were defined a priori for study selection (Textbox 1). The population, intervention, comparator, and outcome strategy was applied to describe the criteria. Only complete and peer-reviewed studies were included. Specific exclusion criteria included partial economic evaluations, systematic reviews or reports, and studies without standard care as a control group. Only studies published in English, Dutch, French, or German were eligible for inclusion. The reference lists of the included studies were searched manually to identify relevant studies. Two reviewers (SR and AP) independently screened the titles and abstracts of all records using Rayyan (Rayyan Systems Inc) [22]. After the initial screening, full texts were retrieved and screened for a second time. The second screening round was independently performed by 2 reviewers (SR and AP). Reasons for exclusion were documented (Figure 1). For both screening rounds, reviewers were blinded from each other’s decision, and disagreements were resolved through discussion.

**Eligibility criteria.**

**Inclusion criteria**

- Intervention
  Cardiac implantable electronic devices: pacemaker, implantable cardioverter-defibrillator, cardiac resynchronization therapy defibrillator, cardiac resynchronization therapy pacemaker, and loop recorder
- Comparator
  Standard care
- Study design
  Complete health economic evaluations (within-trial and model-based)
- Context
  All settings
- Language
  English, French, German, or Dutch

**Exclusion criteria**

- Intervention
  Implantable pulmonary artery pressure monitor
- Study design
  Partial health economic evaluations (outcomes related to costs or effectiveness only)
- Specific criteria
  Systematic reviews, reports, commentaries, congress abstracts, protocols, and animal studies

### Quality Assessment

Two researchers (SR and AP) independently evaluated the original papers using the Consensus Health Economic Criteria (CHEC) checklist to assess the risk of bias [23]. The CHEC checklist included 19 items. Any disagreement was resolved by discussion and consensus. Interpretation of the CHEC list can be found in Multimedia Appendix 3. The included studies were classified into 4 quality categories: excellent (score of 100%), good quality (score between 75% and 100%), moderate quality (score between 50% and 75%), and low quality (score <50%) [24].

### Synthesis of Results

The study characteristics and main outcomes of the original papers are presented in the *Results* section. SR extracted all data. A data extraction sheet was developed using an existing template [17]. The following information was extracted from the included studies: study identification, general study characteristics, results, and authors’ conclusion. The principal outcome measures were health outcomes, cost or income outcomes (eg, the impact on total cost or income, cost or income drivers, cost or income drivers per patient, and cost or income drivers as a percentage of the total cost impact), and incremental cost-effectiveness ratios (ICERs) or cost-utility ratios.

To facilitate comparison across studies, the following adjustments and interpretations were made. First, the cost or income outcomes were presented per patient per year, and different currencies were converted to US Dollar (reference year: 2019 and reference country: United States) [25]. Second, perspectives were categorized into the health care payer perspective, patient perspective, provider perspective, and societal perspective. For the purpose of our study, the provider includes physicians who are directly involved in the care of patients with CIED.

## Results

### Overview

The selection process is shown in Figure 1. From a total of 3305 publications, 15 (0.45%) unique publications were reviewed. Studies were excluded because one of the following reasons: (1) intervention: the paper did not describe telemonitoring patients with a CIED; (2) outcome: the paper contained only a cost analysis and not a cost-effectiveness analysis; and (3) study design or publication: the paper was a partial health economic evaluation, congress abstract, protocol, systematic review, animal study, or with no peer review.

### Population

Characteristics of the included studies can be found in Table 1. All 15 (100%) studies had a primarily male population, except for the Nordland study, which had an almost equal sex distribution (Table 1) [26]. The mean age of the population with pacemakers was between 75 (SD 24.64) and 81 (SD 6.47) years. The mean age of patients with an ICD or CRT-D was between 61 (SD 12.6) and 69 (SD not calculated) years, except for the PREDICT RM study, where >50% of the population was aged >75 years [27]. Furthermore, of the 15 studies, 1 (7%) included only older patients (with a mean age of 81 years) with pacemakers [28], and 2 (13%) ICD or CRT-D studies only included patients with heart failure [11,29].

**Table 1:** Main characteristics of the included studies.

Study	Author and year	Patients, n	Population characteristics	Age (years), mean (SD)	Male participant (%)	CIED^a^ type
Poniente [28]	Bautista-Mesa et al [28], 2022	55	Mean age of 81 years	81 (6.47)	69	Pacemaker
PREDICT RM [27]	Hummel et al [27], 2019	15,254	N/A^b^	53% of participants aged ≥75 years^c^	72	ICD^d^
TARIFF^e^ [30]	Ricci et al [30], 2016	209	N/A	69 (10.17)	85	ICD or CRT-D^f^
Nordland [26]	Lopez-Villegas et al [26], 2020	50	N/A	74.8 (24.64)	52	Pacemaker
EVOLVO^g^ [11]	Zanaboni et al [11], 2013	200	Patients with heart failure	66-69 (SD not reported)	79	ICD or CRT-D
MORE-CARE^h^ [29]	Boriani et al [29], 2016	865	Patients with heart failure	66 (10)	76	CRT-D
Burri et al [31]	Burri et al [31], 2013	N/A	Patients with biventricular CRT-D	65 (SD not reported)	N/A	ICD or CRT-D
Raatikainen et al [32]	Raatikainen et al [32], 2008	41	N/A	62 (10)	83	ICD
Al-Khatib et al [33]	Al-Khatib et al [33], 2009	151	N/A	63 (SD not reported)	72	ICD or CRT-D
CONNECT^i^ [8]	Crossley et al [8], 2011	1997	N/A	65 (12.1)	71	ICD or CRT-D
ECOST^j^ [34]	Guédon-Moreau et al [34], 2014	310	N/A	60.7 (12.6)	90	ICD
EuroEco^k^ [13]	Heidbuchel et al [13], 2015	303	Patients with new or replacement VVI-ICD^l^ or DDD-ICD^m^	62.4 (13.1)	81	ICD or CRT-D
SAVE-HM^n^ trial [35]	Perl et al [35], 2013	115	Patients with dual chamber pacemaker	74 (9)	57	Pacemaker
SAVE-HM^n^ trial [35]	Perl et al [35], 2013	36	Patients with ICD-implant due to primary prevention of sudden cardiac death	62.5 (10)	86	ICD
Chew et al [36]	Chew et al [36], 2020	1830	N/A	66 (SD not reported)	88	ICD or CRT-D
Dario et al [37]	Dario et al [37], 2016	1171	N/A	77.5 (9)	58	Pacemaker
Dario et al [37]	Dario et al [37], 2016	930	N/A	67.5 (12)	79	ICD

^a^CIED: cardiac implantable electronic device.

^b^N/A: not applicable.

^c^Age was a discrete variable in this study (higher of lower than 75 years old).

^d^ICD: implantable cardioverter-defibrillator.

^e^TARIFF: Health Economics Evaluation Registry for Remote Follow-Up.

^f^CRT-D: cardiac resynchronization therapy defibrillator.

^g^EVOLVO: Evolution of Management Strategies of Heart Failure Patients With Implantable Defibrillators.

^h^MORE-CARE: Monitoring Resynchronization Devices and Cardiac Patients.

^i^CONNECT: Clinical Evaluation of Remote Notification to Reduce Time to Clinical Decision.

^j^ECOST: Effectiveness and Cost of ICD Follow-Up Schedule With Telecardiology.

^k^EuroEco: European Health Economic Trial on Home Monitoring in ICD Patients.

^l^VVI-ICD: single-chamber ICD.

^m^DDD-ICD: dual-chamber ICD.

^n^SAVE-HM: Socio-Economic Effects and Cost Saving Potential of Remote Patient Monitoring.

### Study Designs

Tables 2 and 3 show the summary table of results. Of 15 studies, 11 (73%) were conducted in Europe [11,26,28-32,34,35,37], 3 (20%) in the United States [27,33,38], and 1 (7%) in Canada [36]. Of the 15 studies, 3 (20%) calculated the ICER [26-28], 1 (7%) calculated the cost-utility ratio [11], and 11 (73%) calculated the cost impact of telemonitoring. All studies analyzed the health care payer perspective, with 33% (5/15) analyzing the patient perspective [11,28,30,32,34], 13% (2/15) analyzing the societal perspective [33,35], and 13% (2/15) analyzing the provider perspective [13,30].

**Table 2:** Summary of the main results.

Study			Country		Design		Time horizon		CIED^a^ type		Effect		Cost-effectiveness in original currency and in reference year (in US $, 2019)		Conclusion
**Pacemaker studies**															
	Poniente [28]	Spain		Non-RCT^b^		5 years		Pacemaker		QALY^c^ difference: 0.27		ICER^d^: €301.16 per QALY (US $270.09 per QALY)		Cost-effective	
	Nordland [26]	Norway		RCT		1 year		Pacemaker		QALY difference: 0.03		ICER: €53,345 per QALY (US $59.746 per QALY)		Not cost-effective	
	SAVE-HM^e^ trial [35]	Austria		RCT		17 months		Pacemaker		No adverse effects difference		N/A^f^		Cost-saving	
	Dario et al [37], 2016	Italy		Non-RCT		1 year		Pacemaker		Average time reduction to treat patients (−4.1 minutes/follow-up)		N/A		Cost-saving	
**ICD^g^ or CRT-D^h^ studies**															
	PREDICT RM [27]	United States		Real-world		Lifelong		ICD		QALY difference: 0.64		ICER: US $10,752 per QALY (US $12,069 per QALY)		Cost-effective	
	TARIFF^i^ [30]	Italy		Non-RCT		12 months		ICD or CRT-D		QALY difference: 0.02		Not calculated because QALY difference was not significant (*P*=.53)		Cost-saving	
	EVOLVO^j^ [11]	Italy		RCT		16 months		ICD or CRT-D		QALY difference: 0.066^k^ (*P*=.03)		Cost-utility ratio <0		Dominant	
	MORE-CARE^l^ [29]	Europe and Israel		RCT		2 years		CRT-D		QOL^m^ difference:−1		N/A		Cost-saving	
	Burri et al [31], 2013	United Kingdom		Systematic review data		10 years		ICD or CRT-D		Inappropriate shocks: −51%; battery exhaustion: −7%		N/A		Cost-saving	
	Raatikainen et al [32], 2008	Finland		Non-RCT		18 months		ICD		Time burden for patients of −175 minutes and physician of −17 minutes/patient/follow-up		N/A		Cost-effective	
	Al-Khatib et al [33], 2009	United States		RCT		1 year		ICD or CRT-D		EuroQoL difference: 25%; no difference in satisfaction and mortality		N/A		Cost-saving	
	CONNECT^n^ [8]	United States		RCT		15 months		ICD or CRT-D		Time from clinical event to clinical decision: 17.4 days^k^ (*P*<.001)		N/A		Cost-saving	
	ECOST^o^ [34]	France		RCT		27 months		ICD		Physical, psychological, and SF-36^p^ QOL scores: not significant		N/A		Cost-saving	
	EuroEco^q^ [13]	Belgium, Finland, Germany, United Kingdom, Spain, and the Netherlands		RCT		2 years		ICD or CRT-D		SF-36 QOL score: not significant		N/A		Cost-saving	
	SAVE-HM trial [35]	Austria		RCT		26 months		ICD		No adverse effects difference		N/A		Cost-saving	
	Chew et al [36], 2022	Canada		Non-RCT		5 years		ICD or CRT-D		Risk of death (hazard ratio): 0.43^k^ (*P*<.001)		N/A		Cost-saving	
	Dario et al [37], 2016	Italy		Non-RCT		1 year		ICD		Average time reduction to treat patient (−13.7 minute/follow-up)		N/A		Cost-saving	

^a^CIED: cardiac implantable electronic device.

^b^RCT: randomized controlled trial.

^c^QALY: quality-adjusted life year.

^d^ICER: incremental cost-effectiveness ratio.

^e^SAVE-HM: Socio-Economic Effects and Cost Saving Potential of Remote Patient Monitoring.

^f^N/A: not applicable.

^g^ICD: implantable cardioverter-defibrillator.

^h^CRT-D: cardiac resynchronization therapy defibrillator.

^i^TARIFF: Health Economics Evaluation Registry for Remote Follow-Up.

^j^EVOLVO: Evolution of Management Strategies of Heart Failure Patients With Implantable Defibrillators.

^k^The values are statistically significant.

^l^MORE-CARE: Monitoring Resynchronization Devices and Cardiac Patients.

^m^QOL: quality of life.

^n^CONNECT: Clinical Evaluation of Remote Notification to Reduce Time to Clinical Decision.

^o^ECOST: Effectiveness and Cost of ICD Follow-Up Schedule With Telecardiology.

^p^SF-36: The 36-Item Short Form Survey.

^q^EuroEco: European Health Economic Trial on Home Monitoring in ICD Patients.

**Table 3:** Summary table of results related to perspectives and key cost or income drivers.

Study and perspective					Total cost or income^a^ impact compared to standard care in original currency in reference year (US $ pp^b^ per year, 2019) and cost or income^a^ impact drivers			Cost or income^a^ impact drivers pp (US $ pp per year, 2019)		Cost or income^a^ impact drivers as a percentage of total cost or income impact (%)
**Pacemaker studies**										
	**Poniente [28]**									
		**Health care payer perspective**								
				€**, 2012: −€8 (−US $8.96)**						
						Staff costs	−€3.7 (−US $ 4.56)		49	
						Ambulance transport	−€3.2 (−US $3.9)		42	
						Consultation room	−€0.8^c^ (−US $0.9)		10	
		**Patient** **perspective**								
				€**, 2012: −€9 (−US $11.2)**						
						Informal transport	−€5.1^c^ (−US $ 6.2)		58	
						Lost income	−€3.7 (−US $4.58)		42	
	**Nordland [26]**									
		**Health care payer perspective**								
				€**, 2015: €1,808 (US $2,183)**						
						Hospitalization	€1,808.31 (US $2,183)		100	
						Ambulance transport	−€60 (−US $72.5)		−3	
						Physician cost	€39.39^c^(US $47.6)		2	
						Consultation room	€20.17^c^ (US $24.3)		1	
	**SAVE-HM^d^ trial [35]**									
		**Societal** **perspective**								
				€**, 2013: −€914 (−US $1,113)**						
						Transport	−€911.3 (−US $1,020)		99.7	
						Follow-up personnel cost	−€26.7 (−US $32.93)		2	
	**Dario et al [37], 2016**									
		**Health care payer perspective**								
				**€, 2011: −€832^c^ (−US $1,054)**						
						Acute hospitalization	−€816^c^ (−US $1,034)		98	
						Pharmacy medication	−€26 (−US $32.93)		3	
						ED^e^ admission	−€11.89 (−US $15.01)		1	
						Visits and procedure	€22.29 (US $28.22)		−3	
**ICD^f^ or CRT-D^g^ studies**										
	**PREDICT RM [27]**									
		**Health care payer perspective**								
				**US $, 2006: −$566 (−US $635)**						
						Hospitalization	−US $554 (−US $621.94)		98	
						Nonhospital cost	−US $12 (−US $13.44)		2	
	**TARIFF^h^ [30]**									
		**Health care payer perspective**								
				€**, 2011: −€562 (−US $712)**						
						Cardiovascular hospitalization	−€454^c^ (−US $575.1)		80	
						Scheduled visit, protocol based	−€64.24^c^ (−US $81.4)		11	
						Outpatient diagnostic test	−€36.93^c^ (−US $46.82)		7	
						Unscheduled visit	€12.27^c^ (US $15.6)		−2	
						Emergency visit costs	−€15.67 (−US $19.8)		0.03	
						Outpatient clinical evaluation	−€3.12 (−US $3.9)		0.005	
		**Patient** **perspective**								
				€**, 2011: −€68 (−US $86)**						
						Patient loss of work	−€42.34^c^ (−US $53.6)		62	
						Traveling	−€25.86^c^ (−US $32.8)		38	
		**Provider** **perspective**								
				€**, 2011: −€55 (−US $69)**						
						Scheduled visit, protocol based	−€64.24^c^ (−US $81.4)		117	
						Unscheduled visit	€12.27^c^ (US $15.6)		−22	
						Outpatient clinical evaluation	−€3.12 (−US $3.9)		6	
	**EVOLVO^i^ [11]**									
		**Health care payer perspective**								
				€**, 2010: −€167 (−US $219.5)**						
						Hospitalization	−€223 (−US $292.5)		134	
						Scheduled visit, protocol based	−€33.66^c^ (−US $44.1)		20	
						ED^h^ and urgent visit	−€8.81^c^ (−US $11.5)		5	
						Nonurgent in-office visit	€10.68 (−US $14)		−6	
						Diagnostic examinations	−€0.56 (−US $0.78)		0	
						Scheduled remote visit	€32.20^c^ (−US $42.2)		−19	
						Unscheduled remote visit	€56.42^c^ (−US $74)		−34	
		**Patient** **perspective**								
				€**, 2010: −€90 (−US $117)**						
						Scheduled visit, protocol based	−€96.90^c^ (−US $127.6)		110	
						ED and urgent visit	−€23.81^c^ (−US $31.2)		27	
						Nonurgent visit	€30.74 (US $40.32)		−35	
	**MORE-CARE^j^ [29]**									
		**Health care payer perspective**								
				€**, 2014, no reimbursement: −€62.5 (−US $76)**						
						Cardiovascular hospitalization	−€44.3 (−US $53.76)		71	
						Scheduled visit, protocol based	−€37.4 (−US $45.4)		61	
						ED visits	−€0.5 (−US $0.56)		−1	
						Unscheduled visit	€6.4 (US $7.8)		−10	
						Device hospitalization	€13.3 (US $16.1)		−11	
				€**, 2014, with reimbursement: −€44.3 (−US $18)**						
						Cardiovascular hospitalization	−€44.3 (−US $53.8)		306	
						Unscheduled remote check	Maximum −€29.4 (−US $35.7)		203	
						Scheduled remote check	Maximum −€18.6 (−US $22.5)		128	
						Scheduled visit, protocol based	−€37.4 (−US $45.4)		39	
						ED visits	−€0.5 (−US $0.6)		−1	
						Unscheduled visit	€6.4 (US $7.8)		−44	
						Device hospitalization	€13.3 (US $16.1)		−93	
	**Burri et al [31], 2013**									
		**Health care payer perspective**								
				**£, 2007:** **−£3.3 (−US $6.7)**						
						Initial investment and in-hospital follow-up visit	−£3.3 (−US $6.7)		100	
	**Raatikainen et al [32], 2008**									
		**Health care payer perspective**								
				€**, 2006: −€641 (−US $914)**						
						In-office visit^k^, only 1 visit is protocol based	−€560.0 (−US $798.1)		87	
						Traveling^k^	−€198.7 (−US $283.1)		31	
						Remote monitoring^k^	€146.7 (−US $208.9)		−23	
						Accommodation^k^	−€1.3 (−US $1.9)		0	
						Sickness allowance^k^	−€28.0 (−US $39.9)		4	
		**Patient** **perspective**								
				€**, 2006:−€59 (−US $84)**						
						Patient fee^k^	−€58.7 (−US $83.6)		100	
	**Al-Khatib et al [33], 2009**									
		**Societal** **perspective**								
				**US $, 2009: −US $254 (−US $245)**						
						Patient loss of work	−US $383 (−US $370.3)		150	
						Traveling	−US $19 (−US $18.4)		7	
						Follow-up visit, only 1 visit is protocol based	US $148 (US $ 143)		−58	
	**CONNECT^l^ [8]**									
		**Health care payer perspective**								
				**US$, 2008: −US $1,434 (−US $1,243)**						
						Mean cost per hospitalization	−US $1,434.4 (−US $1,243)		100	
	**ECOST^m^ [34]**									
		**Health care payer perspective**								
				€**, 2011: −€927 (−US $1,175)**						
						Cardiovascular hospitalization	−€720 (−US $912.3)		78	
						Device cost	−€533 (−US $675)		58	
						Nonhospital cost	−€227^c^ (−US $287.6)		24	
						Other nonhospital cost	−€182 (−US $230.6)		20	
						Cardiovascular treatment	−€113 (−US $143.1)		12	
						Device management cost	−€74^c^ (−US $93.7)		8	
						ICD ambulatory visit, 3 visits are protocol based	−€40^c^ (−US $50.6)		4	
						Traveling	−€50 (−US $63.4)		0.05	
						Reimbursement	€1000 (US $1,266)		−108	
		**Patient** **perspective**								
				€**, 2011: −€9 (−US $11.2)**						
						Traveling	−€9 (−US $11.4)		100	
	**EuroEco^n^ [13]**									
		**Provider** **perspective**								
				€**, 2013: −€0.5 (−US $1.12)**						
						Follow-up visit, protocol based	−€0.5 (−US $0.56)		100	
		**Health care payer perspective**								
				€**, 2013: −€287 (−US $349)**						
						Hospitalization	−€301 (€326.9)		105	
						Follow-up visit, protocol based	−€0.5 (−US $0.56)		−0.1	
						Examination	€1.5 (US $1.8)		−0.5	
						Other physician visit	€12.5 (US $15.2)		−4	
	**SAVE-HM trial^d^ [35]**									
		**Societal** **perspective**								
				**€, 2013: −€804.9^c^ (−US $981)**						
						Transport	−€787 (−US $959)		98	
						Follow-up personnel cost, protocol based	−€17 (−US $21.3)		2	
	**Chew et al** **[36], 2022**									
		**Health care payer perspective**								
				**CAD $, 2019: −$709.6 (−US $535.4)**						
						Hospitalization	−$682.4^c^ (−US $514.4)		96	
						ED^e^ visits	−$10.2 (−US $7.68)		1	
						In-office visits	−$17 (−US $12.8)		2	
	**Dario** **et al [37], 2016**									
		**Health care payer perspective**								
				€**, 2011: −€338 (−US $428)**						
						Hospitalization	−€295 (−US $374)		96	
						Visits and procedure, protocol based	−€40 (−US $50.6)		12	
						Pharmacy medication	−€31 (−US $39.3)		9	
						Hospital medication	€1.37^c^ (US $1.8)		−0.45	
						ED^e^ admission	€27^c^ (US $34.2)		−8	

^a^If the perspective is health care system or patient, then *cost* and if the perspective is provider, then *income*.

^b^pp: per patient (in health care and patient perspectives) or per physician (in provider perspective).

^c^The values are statistically significant.

^d^SAVE-HM: Socio-Economic Effects and Cost Saving Potential of Remote Patient Monitoring.

^e^ED: emergency department.

^f^ICD: implantable cardioverter-defibrillator.

^g^CRT-D: cardiac resynchronization therapy defibrillator.

^h^TARIFF: Health Economics Evaluation Registry for Remote Follow-Up.

^i^EVOLVO: Evolution of Management Strategies of Heart Failure Patients With Implantable Defibrillators.

^j^MORE-CARE: Monitoring Resynchronization Devices and Cardiac Patients.

^k^Costs were recalculated per patient.

^l^CONNECT: Clinical Evaluation of Remote Notification to Reduce Time to Clinical Decision.

^m^ECOST: Effectiveness and Cost of ICD Follow-Up Schedule With Telecardiology.

^n^EuroEco: European Health Economic Trial on Home Monitoring in ICD Patients.

### Intervention and Comparator

Telemonitoring entailed data transmission and data review. Table 4 shows the frequencies of data transmission, review, and in-office visits of the included studies. In 47% (7/15) of the studies, data were transmitted continuously or daily [26,28,30,31,34,35]; in 20% (3/15) studies, data were transmitted after a device alert [8,11,29]; and in 13% (2/15) studies, data were transmitted every 3 months [32,33]. In 20% (3/15) of the studies, data review was performed daily [28,34,35]; however, in 40% (6/15) of the studies, it was performed after a device alert was received [8,11,26,29-31]. Besides data transmission and review, telemonitoring included scheduled in-office visits. In 33% (5/15) of the studies, all scheduled in-office visits were based on the protocol [11,13,29,30,35]. In 7% (1/15) of the studies, at least 1 scheduled in-office visit was protocol based [37]. In 3 (20%) of the 15 studies, only 1 scheduled in-office visit was protocol based [32-34]. Protocol-based in-office visits are described in Table 4.

**Table 4:** Frequencies of data transmission, review, and in-office visits of included studies.

Study	Brand of CIED^a^	Frequency of data transmission	Frequency of data review	Frequency of in-office visits	
				Telemonitoring	Standard care
Poniente [28]	Medtronic	Continuously	Daily	Mean: 4.4 pp^b^	Mean: 7.5 pp
PREDICT RM [27]	Boston	Not specified	Not specified	Patient dependent	Not specified
TARIFF^c^ [30]	St-Jude	Continuously	After alert and 3 monthly	After 12 months	3 monthly
Nordland [26]	Biotronik	Daily	After alert	Not specified	Not specified
EVOLVO^d^ [11]	Medtronic	After alert and 8 monthly	After alert and 8 monthly	8 monthly	4 monthly
MORE-CARE^e^ [29]	Medtronic	After alert and after 4, 12, and 20 months	After alert and after 4, 12, and 20 months	8 monthly	4 monthly
Burri et al [31]	Biotronik	Daily	After alert	12 monthly	4 monthly
Raatikainen et al [32]	Medtronic	After 3 and 6 months	After 3 and 6 months	After 9 months	6 monthly
Al-Khatib et al [33]	Medtronic	3 monthly	3 monthly	After 6 months through telephone and after 12 months	3 monthly
CONNECT^f^ [8]	Medtronic	After alert and 3 monthly	After alert and 3 monthly	After 1 month and 12 months	3 monthly
ECOST^g^ [34]	Biotronik	Daily	Daily	After 1 to 3 months and thereafter 12 monthly	After 1 month to 3 months and thereafter 6 monthly
EuroEco^h^ [13]	Biotronik	Continuously	Depending on the researcher	After 6 weeks and thereafter 12 monthly	After 6 weeks, thereafter 12 monthly, and planned visits depending on the hospital
SAVE-HM^i^ trial [35]	Biotronik pacemaker	Daily	Daily	No	12 monthly
SAVE-HM trial [35]	Biotronik ICD^j^	Daily	Daily	12 monthly	6 monthly
Chew et al [36]	N/A^k^	N/A	N/A	N/A	N/A
Dario et al [37]	Biotronik, Medtronic, Boston, St-Jude, and Sorin Group pacemaker	Continuously	Daily	Not unless necessary	12 monthly
Dario et al [37]	Biotronik, Medtronic, Boston, St-Jude, and Sorin Group ICD	Continuously	Daily	At least 1	6 monthly

^a^CIED: cardiac implantable electronic device.

^b^pp: per patient.

^c^TARIFF: Health Economics Evaluation Registry for Remote Follow-Up.

^d^EVOLVO: Evolution of Management Strategies of Heart Failure Patients With Implantable Defibrillators.

^e^MORE-CARE: Monitoring Resynchronization Devices and Cardiac Patients.

^g^ECOST: Effectiveness and Cost of ICD Follow-Up Schedule With Telecardiology.

^h^EuroEco: European Health Economic Trial on Home Monitoring in ICD Patients.

^i^Save-HM: Socio-Economic Effects and Cost Saving Potential of Remote Patient Monitoring.

^j^ICD: implantable-cardioverter defibrillator.

^k^N/A: not applicable.

### Effectiveness

Effectiveness results of telemonitoring can be found in Table 2. Of the 15 studies, 9 (60%) investigated a quality-adjusted life year (QALY) or quality of life (QOL) difference [11,26-30,33,34]. A total of 53% (8/15) of studies reported an increase in QALY or QOL [11,26-28,30,33,34,39], but the QALY or QOL increase was only statistically significant in 1 (13%; *P*=.03) of the 8 studies [26-28,30,33,34]. In contrast, only 1 (11%) of the 9 studies investigating QOL or QALY reported a significant decrease in QOL [29]. Comparing all studies, QALY differences ranged from 0.03 to 0.27 in patients with pacemakers and ranged from −1 to 0.64 in patients with ICD or CRT-D.

Besides QALY or QOL, several studies reported other health outcomes. Chew et al [36] indicated that the risk of death was lower with telemonitoring. Al-Khatib et al [33] reported that mortality and general patient satisfaction with telemonitoring were equal to those of standard care. Crossley et al [8] reported that the time between the clinical event and the clinical decision was 17.4 days shorter in patients with an ICD or CRT-D on telemonitoring than in those on standard care (*P*<.001). Burri et al [31] indicated that telemonitoring patients with ICD or CRT-D led to fewer inappropriate shocks (−51%) and a reduction in battery exhaustion (−7%). Raatikainen et al [32] indicated that telemonitoring patients with an ICD reduced the average total time spent on device follow-up, with 17 minutes per patient per follow-up for physicians and 175 minutes per patient per follow-up for patients. Similarly, Dario et al [37] indicated that the time spent by physicians to treat the patient reduced by an average of 4.1 minutes per follow-up in patients with pacemakers and an average of 13.7 minutes per follow-up in patients with an ICD (SD was not reported).

### Economic Impact

The results of the economic impact of telemonitoring are presented in Table 2. Of the 15 studies, 4 (27%) investigated the cost impact of telemonitoring in patients with pacemakers [26,28,35,37]. From a health care payer perspective, 1 (25%) of the 4 pacemaker studies indicated that telemonitoring increased costs with US $2183 per patient per year (not statistically significant) mainly because of increased hospitalization costs [26]. A total of 2 (50%) of the 4 pacemaker studies indicated that telemonitoring reduced costs by US $8.9 and US $1054 per patient per year mainly because of a reduction in hospitalization and staff costs, respectively [28,37]. Therefore, hospitalizations reduced costs in the study by Dario et al [37] but increased costs in the study by Lopez-Villegas et al [26]. From a patient and societal perspective, the results indicated that telemonitoring reduced costs by US $11 and US $1113 per patient per year, respectively, mainly because of lower transport costs [28,35].

Of the 15 studies, 13 (87%) investigated the cost or income impact of telemonitoring in patients with an ICD or CRT-D [8,11,13,27,29-37]. A total of 11 (85%) of the 13 ICD or CRT-D studies investigated the cost impact of telemonitoring from a health care payer perspective, all indicating that telemonitoring reduced costs for patients with an ICD or CRT-D [8,11,13,27,29-32,34,36,37]. A total of 9 (82%) of the 11 health care payer perspective studies indicated that hospitalization was the largest driver for cost reduction for patients with an ICD or CRT-D [8,11,13,27,29,30,34,36,37]. The hospitalization cost reduced by up to US $912.3 per patient per year [34]. In addition, scheduled in-office visits were reported as a driver for cost reduction in 5 (45%) of the 11 health care payer perspective studies, as up to 61% of the total cost reduction was due to a decrease in the number of scheduled in-office visits [11,29,30,32,34]. Besides cost drivers that reduced costs, there were also drivers that increased costs. In 3 (27%) of the 11 health care payer perspective studies, unscheduled visits increased the total cost impact of telemonitoring [11,13,29,30,33]. A total of 3 (20%) of the 15 studies indicated that the cost reduction for scheduled in-office visits outweighed the cost increase for unscheduled in-office visits (−US $81.4 vs US $15.6, −US $45.4 vs US $7.8, and −US $44.1 vs US $14/patient/year) [11,29,30].

The results of 4 (31%) of the 13 ICD or CRT-D studies that investigated the cost impact of telemonitoring from the patients’ perspective [11,30,32,34] indicated that patient and caregiver loss of work or activity [30], scheduled in-office visits [11], and transport [34] were the largest drivers for cost reduction. The results of 2 (15%) of the 13 ICD or CRT-D studies that investigated the income impact of telemonitoring from a provider perspective indicated that the loss of reimbursed (scheduled) in-office visits was the most important factor for income loss due to telemonitoring [13,30], reducing income by up to €72.7 (US $77.21) per patient per year [30].

### ICER and Cost-Utility Ratio

Results on ICER and the cost-utility ratio are presented in Table 2. Of the 15 studies, 3 (20%) calculated the ICER from a health care payer perspective [26-28] and 1 (7%) calculated the cost-utility ratio from a health care payer perspective [11]. Of the 15 studies, 2 (13%) calculating ICER were conducted with patients with pacemakers [26,28]. Notably, of the 2 studies, 1 (50%) indicated that telemonitoring was cost-effective (ICER: US $270.09/QALY) [28], and 1 (50%) indicated that telemonitoring was not cost-effective (ICER: US $64,410/QALY) [26]. For patients with an ICD or CRT-D, of the 2 studies, 1 (50%) indicated that telemonitoring was cost-effective (ICER: US $12,069/QALY) [27] and 1 (50%) indicated that telemonitoring was dominant [11].

### Critical Appraisal

The critical appraisal of the individual studies is provided in Tables 5 and 6. Of the 15 studies, 1 (7%) was classified as excellent (score of 100%) [13], 8 (53%) had a good quality score (100%<score>75%) [26,28,30,31,33,34,36,37], and 6 (40%) had a moderate quality score (75%<score>50%) [8,11,27,29,32,35]. A total of 3 (20%) of the 15 studies scored the lowest, with 59% each [8,29,32]. More than 50% (>8/15) of the studies scored low for the items *cost valuation* (item 9) [11,27-32,34,35,37], *discounting* (item 14) [8,11,29,30, 32,34,35,37], and *no conflict of interest* (item 18) [8,11,27, 29,30,32,34,35]. All studies scored high on the items *study population* (item 1), *study design* (item 4), *time horizon* (item 10), *outcome identification* (item 11), *outcome measurement* (item 12), and *ethics* (item 19).

**Table 5:** Quality assessment of the first 8 studies.

	EuroEco^a^ [13]	Nordland [26]	Poniente [28]	Burri et al [31]	Al-Khatib et al [33]	Chew et al [36]	TARIFF^b^ [30]	ECOST^c^ [34]
Study population	✓^d^	✓	✓	✓	✓	✓	✓	✓
Competing alternatives	✓	X^e^	✓	✓	✓	X	✓	✓
Research question	✓	✓	X	✓	X	✓	✓	✓
Study design	✓	✓	✓	✓	✓	✓	✓	✓
Time horizon	✓	✓	✓	✓	✓	✓	✓	✓
Perspective	✓	✓	✓	✓	✓	✓	✓	✓
Cost identification	✓	✓	✓	X	✓	✓	✓	✓
Cost measurement	✓	✓	✓	✓	✓	✓	✓	✓
Cost valuation	✓	✓	X	X	✓	✓	X	X
Outcome identification	✓	✓	✓	X	✓	✓	✓	✓
Outcome measurement	✓	✓	✓	✓	✓	✓	✓	✓
Outcome valuation	✓	✓	✓	N/A^f^	N/A	N/A	✓	N/A
Incremental analysis	N/A	✓	✓	N/A	N/A	N/A	N/A	N/A
Discounting	✓	N/A	✓	✓	✓	✓	X	X
Sensitivity analysis	✓	✓	✓	✓	X	✓	✓	✓
Conclusions	✓	✓	✓	✓	✓	✓	✓	✓
Generalizability	✓	✓	✓	✓	✓	X	✓	✓
No conflicts of interest	✓	✓	✓	✓	✓	✓	X	X
Ethics	✓	✓	✓	✓	✓	✓	✓	✓
Values, n (%)	18 (100)	16 (94)	15 (89)	15 (88)	15 (88)	15 (88)	15 (83)	14 (82)

^a^EuroEco: European Health Economic Trial on Home Monitoring in ICD patients.

^b^TARIFF: Health Economics Evaluation Registry for Remote Follow-Up.

^c^ECOST: Effectiveness and Cost of ICD Follow-Up Schedule With Telecardiology.

^d^Sufficient attention was given to this aspect.

^e^Insufficient attention was given to this aspect.

^f^N/A: not applicable.

**Table 6:** Quality assessment of the last 7 studies.

	Dario et al [37]	PREDICT RM [27]	SAVE-HM^a^ [35]	EVOLVO^b^ [11]	Raatikainen et al [32]	MORE-CARE^c^ [29]	CONNECT^d^ [8]
Study population	✓^e^	✓	✓	✓	✓	✓	✓
Competing alternatives	✓	X^f^	✓	✓	✓	✓	✓
Research question	✓	X	X	X	✓	X	X
Study design	✓	✓	✓	✓	✓	✓	✓
Time horizon	✓	✓	✓	✓	✓	✓	✓
Perspective	✓	✓	✓	✓	X	✓	X
Cost identification	✓	X	X	✓	X	X	X
Cost measurement	✓	✓	✓	X	✓	X	X
Cost valuation	X	X	X	X	X	X	✓
Outcome identification	✓	✓	✓	✓	✓	✓	✓
Outcome measurement	✓	✓	✓	✓	✓	✓	✓
Outcome valuation	N/A^g^	✓	N/A	✓	N/A	N/A	N/A
Incremental analysis	N/A	✓	N/A	N/A	N/A	N/A	N/A
Discounting	X	✓	X	X	X	X	X
Sensitivity analysis	X	✓	✓	✓	X	X	✓
Conclusions	✓	✓	✓	✓	X	✓	✓
Generalizability	✓	✓	✓	X	✓	✓	X
No conflicts of interest	✓	X	X	X	X	X	X
Ethics	✓	✓	✓	✓	✓	✓	✓
Values, n (%)	14 (82)	14 (74)	12 (71)	12 (67)	10 (59)	10 (59)	10 (59)

^a^Save-HM: Socio-Economic Effects and Cost Saving Potential of Remote Patient Monitoring.

^b^EVOLVO: Evolution of Management Strategies of Heart Failure Patients With Implantable Defibrillators.

^c^MORE-CARE: Monitoring Resynchronization Devices and Cardiac Patients.

^d^CONNECT: Clinical Evaluation of Remote Notification to Reduce Time to Clinical Decision.

^e^Sufficient attention is given to this aspect.

^f^Insufficient attention is given to this aspect.

^g^N/A: not applicable.

## Discussion

### Principal Findings and Comparison With Prior Work

The primary aim of this study was to investigate the cost-effectiveness of telemonitoring patients with an ICD or CRT-D and a pacemaker from different perspectives.

From a health care payer perspective, most studies indicated that telemonitoring was a cost-saving and effective alternative to standard care. The most important driver for cost reduction was hospitalizations, both in patients with a pacemaker and those with an ICD or CRT-D. The cost of hospitalizations was reduced by up to US $912.3 per patient per year [34]. Moreover, the reduction of scheduled in-office visits was the second most important cost-saving factor in most ICD or CRT-D studies, with up to 61% of the total cost reduction. Previous research indicated that up to 55% of the device follow-ups were routine checks with no actionable events or device programming [35,40,41]. Several researchers pointed out that most scheduled in-office visits could be replaced by telemonitoring without affecting the quality of care [7,34] and with potentially diagnosing >99.5% of arrhythmia and device problems [41]. Although scheduled in-office visits decreased, our results show that unscheduled in-office visits increased because of telemonitoring patients with an ICD or CRT-D, probably because of the possible faster detection of arrhythmia and device malfunction by telemonitoring [8]. However, in all studies analyzing both scheduled and unscheduled in-office visits, the cost reduction for scheduled in-office visits outweighed the cost increase for unscheduled in-office visits [11,29,30].

From a patient perspective, our results indicated that the reduction of professional activity, transport time, and costs due to scheduled in-office visits are the most important factors for cost reduction.

The provider perspective was investigated less frequently in the included studies, although it is very relevant. Owing to the reduction of scheduled in-office visits, providers will lose income with telemonitoring if no reimbursement exists for telemonitoring but only for in-office visits. As a result, providers will be stimulated to maintain the classic follow-up instead of telemonitoring. Of the 15 studies, 1 (7%) observed that the total cost for insurance payers does not increase in countries where telemonitoring is reimbursed [13]. As telemonitoring decreases the overall costs from a health care payer perspective, there is room for proper compensation for providers to transition from in-office care to remote care. Hence, correct compensation (which is possible while still saving on the overall health care cost) will stimulate providers to switch to telemonitoring as the desired care path for patients with a CIED.

All studies reported the effectiveness of telemonitoring. Of the 15 studies, 9 (60%) indicated a QALY or QOL difference. Furthermore, 89% (8/9) of these studies indicated an increase in QALYs or QOL for telemonitoring patients with pacemakers or ICD or CRT-D, ranging from −1 to 0.64. Some studies (3/9, 33%) indicated this QALY or QOL increase was the result of the reduced routine in-office visits [7,34]. However, the QALY or QOL increase was only statistically significant (and positive) in 1 (11%) of the 9 studies [11]. Nevertheless, patient questionnaires have demonstrated a high acceptance of telemonitoring among patients with pacemakers and those with ICDs [39]. Moreover, telemonitoring is reported to lead to an increased sense of security [39]. Furthermore, the results indicated that telemonitoring leads to fewer inappropriate shocks, an important determinant of QALY, in patients with an ICD or CRT-D [31].

The cost-effectiveness analyses may be sensitive to the heterogeneity among the organization of telemonitoring in different hospitals. This may include different devices, the number of transmissions, the configuration of alerts, and hospital visit scheduling [26]. It seems reasonable to expect that the efficiency of telemonitoring not only depends on the technology but also on the organization of the service. If hospitals see telemonitoring as an additional service, on top of standard care, less cost-savings may be seen than if hospitals see telemonitoring as a substitute for standard care. A radical organizational change could lead to larger cost-savings, as suggested by an observational study by Facchin et al [42]. Moreover, such radical change may include a strategy involving other physicians, such as general practitioners, and referring cardiologists, that is, an integrated health care delivery [37].

Furthermore, the comparison between studies is challenged by differences in study design. The Poniente study by Bautista-Mesa et al [28] followed up patients with pacemakers for 12 months and indicated a QALY increase of 0.09 for telemonitoring. However, after 5 years of follow-up, the results indicated a QALY decrease of 0.20 for telemonitoring. Bautista-Mesa et al [28] indicated that some of the telemonitoring benefits (eg, reduction of in-office visits) may not be appreciated in the long term. Therefore, the evolution of utilities may be different depending on the follow-up time. In addition, the results indicated that hospitalizations reduced costs in the study by Dario et al [37] but increased costs in the study by Lopez-Villegas et al [26]. This discrepancy might be explained because significantly fewer patients were included in the study by Lopez-Villegas et al (50 vs 2101 patients). None of the 25 patients in the conventional follow-up group were hospitalized, whereas 12% (3/25) of the patients were hospitalized in the remotely monitored group (all for pacemaker problems) [26]. Furthermore, the included studies relied disproportionally on male participants, except for the Nordland study [26]. This may be explained by the significant sex disparity in ICD implantation rates, pointed out by Ingelaere et al [43]. Ingelaere et al [43] could not completely explain these differences by prevalence differences of cardiomyopathies and imply a possible undertreatment of women. Another study [44] observed an undertreatment of women with coronary heart disease, as they are less likely to undergo coronary angiography. Therefore, men may undergo more expensive treatments than women. This can explain why the included cost-effectiveness studies may present an overly positive result. In addition, time differences may impact the quality and cost-effectiveness of telemonitoring, as telemonitoring may evolve over time. However, our results did not provide meaningful insights in this respect.

The cost-effectiveness analyses may be sensitive to the heterogeneity among health care systems. From a provider perspective, our results indicated that telemonitoring generates lesser profit than standard care in the absence of reimbursement. Therefore, the lack of reimbursement is generally perceived as a major implementation barrier to telemonitoring, affecting 80% of the centers [45]. Consequently, providers tend to continue with standard care instead of telemonitoring. However, from a health care payer perspective, our results indicated that telemonitoring was still cost-saving even with reimbursement [13,34]. To stimulate providers to use telemonitoring, provider compensation should be provided based on overall health care cost-savings, making telemonitoring possible if it is preferred as the way to deliver CIED follow-up care.

### Limitations

Because of the large discrepancies between health care systems’ organization, costs, access, delivery, quality, and reimbursement of cardiac care, any generalization may be perceived as inaccurate [37,46]. For instance, the included studies were mainly performed in Western countries. The results may not be generalizable to non-Western countries. Therefore, the cost-effectiveness results are contingent on the context in which they were analyzed [46]. Another limitation of this research is that 40% (6/15) of the included studies are not randomized controlled trials. These studies may have unobserved confounding factors that cannot be controlled for. Finally, cost analyses were excluded in this study because of our research objective. However, future cost analyses could draw a lot of information from analyzing these excluded studies.

### Conclusions

Telemonitoring patients with CIED may be a cost-effective alternative to standard follow-up. Moreover, telemonitoring may lead to a cost reduction from a health care and patient perspective, mainly by the reduction of hospitalizations and scheduled in-office visits. Owing to the reduction in scheduled in-office visits, providers’ income tends to decrease when implementing telemonitoring without proper reimbursement. Introducing appropriate reimbursement could make telemonitoring sustainable for providers, while still being cost-effective from a health care payer perspective.

## Appendix

Multimedia Appendix 1 PRISMA-ScR (Preferred Reporting Items for Systematic Reviews and Meta-Analyses extension for Scoping Reviews) checklist.

Multimedia Appendix 2 Search strategy.

Multimedia Appendix 3 Interpretation Consensus Health Economic Criteria list.

## Abbreviations

CHEC: Consensus Health Economic Criteria
CIED: cardiac implantable electronic device
CRT-D: cardiac resynchronization therapy defibrillator
ICD: implantable cardioverter-defibrillator
ICER: incremental cost-effectiveness ratio
PRISMA: Preferred Reporting Items for Systematic Reviews and Meta-Analyses
PRISMA-ScR: Preferred Reporting Items for Systematic Reviews and Meta-Analyses extension for Scoping Reviews
PROSPERO: International Prospective Register of Systematic Reviews
QALY: quality-adjusted life year
QOL: quality of life

## Data Availability

All data generated or analyzed during this study are included in this published article and its multimedia appendices.
