# Supplementary material for: Investigating the Cost-Effectiveness of Telemonitoring Patients With Cardiac Implantable Electronic Devices: Systematic Review
Source: J Med Internet Res. 2024 Apr 19;26:e47616. doi: 10.2196/47616 (PMC11069092; doi:10.2196/47616)
Supplement: Multimedia Appendix 2 [file jmir_v26i1e47616_app2.docx]

**Multimedia Appendix 2**

***Investigating the Cost-Effectiveness of Telemonitoring Patients With Cardiac Implantable Electronic Devices: Systematic Review***

*Authors*: Sarah Raes^*^, Andrea Prezzi, Rik Willems, Hein Heidbuchel, Lieven Annemans

*Corresponding author:

Department of Public Health and Primary Care, Ghent University, Ghent, Belgium [sarah.raes@ugent.be](mailto:sarah.raes@ugent.be)

***Search string PubMed***

(“Pacemaker, Artificial”[Mesh] OR “pacemaker”[All Fields] OR “pacemaker follow-up”[All Fields] OR “heart failure”[All Fields] OR “implantable devices”[All Fields] OR “cardiovascular implantable electronic devices”[All Fields] OR “Defibrillators, Implantable”[Mesh] OR “pacemaker”[All Fields] OR “Cardiac implantable electronic devices”[All Fields] OR “implantable electronic cardiac devices”[All Fields] OR “Invasive monitoring”[All Fields] OR “remote cardiac monitoring”[All Fields] OR “cardiology”[All Fields] OR “telecardiology”[All Fields] OR “heart disease”[All Fields] OR “heart failure management”[All Fields] OR “Implantable cardioverter defibrillator”[All Fields] OR “ICD follow-up”[All Fields] OR “ICD”[All Fields])

AND

(“telemonit*”[All Fields] OR “remote monitoring”[All Fields] OR “remote patient monitoring”[All Fields] OR “home telemonitoring”[All Fields] OR “remote follow-up”[All Fields] OR “home telemonitoring”[All Fields] OR “remote medicine”[All Fields] OR “Telemedicine”[Mesh] OR “telemedicine”[All Fields] OR “digital health”[All Fields] OR “telehealth”[All Fields] OR “ehealth”[All Fields] OR “e-health”[All Fields] OR “mhealth”[All Fields] OR “mobile health”[All Fields] OR “apps”[All Fields] OR “web”[All Fields] OR “webbased”[All Fields] OR “internet”[All Fields] OR “information technology”[All Fields])

AND

(“Cost-Benefit Analysis”[Mesh] OR “cost effectiveness”[All Fields] OR “cost-effectiveness”[All Fields] OR “Cost-Benefit Analysis”[All Fields] OR “cost benefit analysis”[All Fields] OR “cost effectiveness analysis”[All Fields] OR “cost-effectiveness analysis”[All Fields] OR “incremental cost effectiveness ratio”[All Fields] OR “cost utility analysis”[All Fields] OR “cost-utility”[All Fields] OR “cost-utility analysis”[All Fields] OR “cost utility”[All Fields] OR “cost minimization analysis”[All Fields] OR “quality adjusted life year”[All Fields] OR “quality of life”[All Fields] OR “Quality-adjusted life year”[All Fields] OR “health economic evaluation”[All Fields] OR “costs”[All Fields] OR “economic impact”[All Fields] OR “economic analysis”[All Fields])

***Search string Embase***

(‘cardiac rhythm management device’/exp OR ‘defibrillator’/exp OR ‘cardiac implantable electronic

device’/exp OR ‘heart disease’/exp OR ‘cardiology’/exp) AND (‘telemonitoring’/exp OR ‘telecardiology’/exp OR ‘mhealth’/exp OR ‘home monitoring’/exp OR ‘patient monitoring’/exp OR ‘medical informatics’/exp) AND (‘economic evaluation’/exp OR ‘incremental cost effectiveness ratio’/exp OR ‘quality adjusted life year’/exp)

***Search string Web of Science***

TS=((“Pacemaker, Artificial” OR “pacemaker” OR “pacemaker follow-up” OR “heart failure” OR “implantable devices” OR “cardiovascular implantable electronic devices” OR “Defibrillators, Implantable” OR “pacemaker” OR “Cardiac implantable electronic devices” OR “implantable electronic cardiac devices” OR “Invasive monitoring” OR “remote cardiac monitoring” OR “cardiology” OR “telecardiology” OR “heart disease” OR “heart failure management” OR “Implantable cardioverter defibrillator” OR “ICD follow-up” OR “ICD”) AND (“telemonit*” OR “remote monitoring” OR “remote patient monitoring” OR “home telemonitoring” OR “remote follow-up” OR “home telemonitoring” OR “remote medicine” OR “Telemedicine” OR “telemedicine” OR “digital health” OR “telehealth” OR “ehealth” OR “e-health” OR “mhealth” OR “mobile health” OR “apps” OR “web” OR “web-based” OR “internet” OR “information technology”) AND (“Cost-Benefit Analysis” OR “cost effectiveness” OR “cost-effectiveness” OR “Cost-Benefit Analysis” OR “cost benefit analysis” OR “cost effectiveness analysis” OR “cost-effectiveness analysis” OR “incremental cost effectiveness ratio” OR “cost utility analysis” OR “cost-utility” OR “costutility analysis” OR “cost utility” OR “cost minimization analysis” OR “quality adjusted life year” OR “quality of life” OR “Quality-adjusted life year” OR “health economic evaluation” OR “costs” OR “economic impact” OR “economic analysis”))

***Search string EconLit:***

(“pacemaker” OR “defibrillator” OR “ICD” OR “cardiac device” OR “cardiac implantable electronic device”) AND (“remote monitoring” OR “home monitoring” OR “telemonitoring” OR “patient monitoring”)
