# Supplementary material for: Investigating the Cost-Effectiveness of Telemonitoring Patients With Cardiac Implantable Electronic Devices: Systematic Review
Source: J Med Internet Res. 2024 Apr 19;26:e47616. doi: 10.2196/47616 (PMC11069092; doi:10.2196/47616)
Supplement: Multimedia Appendix 3 [file jmir_v26i1e47616_app3.docx]

**Multimedia Appendix 3**

***Investigating the Cost-Effectiveness of Telemonitoring Patients With Cardiac Implantable Electronic Devices: Systematic Review***

*Authors*: Sarah Raes^*^, Andrea Prezzi, Rik Willems, Hein Heidbuchel, Lieven Annemans

*Corresponding author:

Department of Public Health and Primary Care, Ghent University, Ghent, Belgium [sarah.raes@ugent.be](mailto:sarah.raes@ugent.be)

***Interpretation CHEC list***

| **Item** | **Question** | **Assessment instruction** | **Interpretation of the instructions and/or adaptations** |
| --- | --- | --- | --- |
| 1 | Is the study population clearly described? | The relevant clinical characteristics, entry and eligibility criteria, as well as drop-out during follow-up should be stated explicitly. |  |
| 2 | Are competing alternatives clearly described? | A detailed description should be given of the competing interventions. This should encompass a clear and specific statement of the primary objective of each alternative, as well as relevant factors, such as intensity, duration, and frequency. | Standard care should be clearly described (e.g. how many in-office visits). |
| 3 | Is a well-defined research question posed in answerable form? | A research question has to identify clearly the alternatives being compared and the population for which the comparison is made. | A sentence should be written with a description of the population, the intervention and the control group treatment, and the aim (outcome). |
| 4 | Is the economic study design appropriate to the stated objective? | An appropriate economic study design is a full economic evaluation (comparison of costs and effects of 2 or more interventions) based on primary research (cohort, case-control, randomized controlled trial). |  |
| 5 | Is the chosen time horizon appropriate in order to include relevant costs and consequences? | The period of analysis of the study is the time horizon. This time horizon should always be equal for costs and outcomes if these are combined in a ratio. The time span should be long enough to include all relevant costs and outcomes relating the intervention. Ideally, the follow-up period should be extended till the situation is stabilized with reference to costs and effects. | Time horizon should be at least 1 year. |
| 6 | Is the actual perspective chosen appropriate? | ‘Perspective’ indicates from which point of view an economic evaluation study is performed. If the study is performed from a societal perspective tick ‘yes’, as all relevant costs and consequences of an interventions and disease are taken into account, if possible. Other narrower perspectives will only include certain components. The authors should motivate why a narrower perspective is valid. |  |
| 7 | Are all important and relevant costs for each alternative identified? | A full identification of all important and relevant costs should be given in relation to the perspective and the research question. |  |
| 8 | Are all costs measured appropriately in physical units? | The costs should be measured appropriately in physical units. The instrument by which the costs are measured should be valid and clearly stated (e.g. interview, questionnaire, cost-diary). |  |
| 9 | Are costs valued appropriately? | The sources of valuation should be clearly stated for each cost price of every volume parameter and their reference year. The main cost should be calculated based on depleted sources, no tariffs should be used. | The cost unit must be clearly stated (e.g. €, $) with the reference year. |
| 10 | Are all important and relevant outcomes for each alternative identified? | A full identification of all important and relevant outcomes should be given in relation to the perspective and the research question. | E.g. QALY |
| 11 | Are all outcomes measured appropriately? | The outcome measurement should result from the outcome identification and this should be straightforward (e.g. if mortality is a main outcome measure this should be taken into account in the analysis). The instrument by which the outcomes are measured should be valid and clearly stated. | E.g. EQ-5D  When the ICER is calculated, the corresponding threshold should also be mentioned for interpreting the ICER. |
| 12 | Are outcomes valued appropriately? | The method of outcome valuation should be clearly stated. Examples of valuation methods are Discrete Choice Experiments (e.g. Conjoint analysis, Contingent valuation), Direct utility assessment (VAS, TTO, SG, etc.), Indirect utility assessment (HUI, EQ-5D, QWB, etc.), Person trade off, etc. | This item is only applicable in studies that used quality- or disability-adjusted life years (QALYs or DALYs). Otherwise, this item was scored “not applicable”. |
| 13 | Is an incremental analysis of costs and outcomes of alternatives performed? | An incremental analysis should examine the additional costs from one intervention over another, compared to the additional outcomes that it delivers. The incremental costs-effectiveness ratio is obtained by dividing the costs differences (C2-C1) by the outcome differences (O2-O1) for the alternatives. | This item is scored “not applicable” when the ICER or cost-utility ratio was not calculated. |
| 14 | Are all future costs and outcomes discounted appropriately? | Discounting is done appropriately if all costs and outcomes are converted to one single year, based on a motivated discount rate. | This item is scored “not applicable” for studies of one year or less. |
| 15 | Are all important variables, whose values are uncertain, appropriately subjected to sensitivity analysis? | All variables in the analysis are potential candidates for the sensitivity analysis. Only variables that are certain or which have a minimal impact on the study results (based on the preliminary analysis) can be excluded from the sensitivity analysis. Furthermore, a justification should be given over the range of the variables used in the sensitivity analysis. | In studies only calculating cost outcomes, the mean and standard deviation or confidence interval should be presented. |
| 16 | Do the conclusions follow from the data reported? | Do the authors interpret their results cautiously and are their conclusions justified by the data. |  |
| 17 | Does the study discuss the generalizability of the results to other settings and patient/client groups? | This can be done by being explicit about the viewpoint of analysis and by indicating how particular costs and outcomes vary by location, setting, patient population, care provider, etc. |  |
| 18 | Does the article indicate that there is no potential conflict of interest of study researcher(s) and funder(s)? | If an external agency finances the study, a statement should explicitly be given about who finances the study to guarantee transparency in the relationship between the sponsor and the researcher. Whenever a potential conflict of interest is possible a declaration should be given of ‘competing interest’. |  |
| 19 | Are ethical and distributional issues discussed appropriately? | Does the article notes ethical aspects and elaborates on the characteristics of the population experiencing the disease or the intervention (young, old, poor, wealthy) and how this may have distributional implications. |  |
